# Assessment of Children’s Exposure to Intelligent Transport System 5.9 GHz Vehicular Connectivity Using Numerical Dosimetry

**DOI:** 10.3390/s23115170

**Published:** 2023-05-29

**Authors:** Martina Benini, Marta Parazzini, Marta Bonato, Silvia Gallucci, Emma Chiaramello, Serena Fiocchi, Gabriella Tognola

**Affiliations:** 1 Department of Electronics, Information and Bioengineering (DEIB), Politecnico di Milano, Piazza Leonardo da Vinci 32, 20133 Milan, Italy; silvia.gallucci@ieiit.cnr.it; 2 Cnr-Istituto di Elettronica e di Ingegneria dell’Informazione e delle Telecomunicazioni, Piazza Leonardo da Vinci 32, 20133 Milano, Italy; marta.parazzini@ieiit.cnr.it (M.P.); marta.bonato@ieiit.cnr.it (M.B.); emma.chiaramello@ieiit.cnr.it (E.C.); serena.fiocchi@ieiit.cnr.it (S.F.); gabriella.tognola@ieiit.cnr.it (G.T.)

**Keywords:** RF exposure assessment, road user, V2X, intelligent transport system, vehicular connectivity

## Abstract

This study investigates the radio-frequency electromagnetic field exposure (RF-EMF) levels in pedestrians generated by vehicular communication technology. We specifically investigated exposure levels in children of different ages and both genders. This study also compares the children’s exposure levels generated by such technology with those of an adult investigated in our previous study. The exposure scenario consisted of a 3D-CAD model of a vehicle equipped with two vehicular antennas operating at 5.9 GHz, each fed with 1 W power. Four child models were analyzed near the front and back of the car. The RF-EMF exposure levels were expressed as the Specific Absorption Rate (SAR) calculated over the whole body and 10 g mass (SAR_10g_) of the skin and 1 g mass (SAR_1g_) of the eyes. The maximum SAR_10g_ value of 9 mW/kg was found in the skin of the head of the tallest child. The maximum whole-body SAR was 0.18 mW/kg and was found in the tallest child. As a general result, it was found that children’s exposure levels are lower than those of adults. All the SAR values are well below the limits recommended by the International Commission on Non-Ionizing Radiation Protection (ICNIRP) in the general population.

## 1. Introduction

In the last decade, the number of vehicles circulating on the roads exhibited a quick year-by-year increase. In addition to the many advantages, the increase in vehicles on the roads comes with several drawbacks, such as increases in deaths on roads and traffic congestion, all of which imply an increase in CO_2_ emissions [1]. For this reason, nowadays, the concept of on-road driving is forcing the development of innovative vehicle connectivity technologies to improve road safety and manage traffic congestion through real-time traffic information shared among the cars and the road infrastructure. Car connectivity is also the backbone of the forthcoming fully autonomous driving. Much research has been conducted over the past years for the development of a new mobility concept named Intelligent Transport Systems (ITSs) [2,3]. ITSs deeply rely on ad hoc wireless communication technologies called “vehicle-to-everything-communication” (V2X) and on technologies for sensing the environment outside the vehicle (through Advanced Driver Assistance Systems (ADAS)) and inside the vehicle (through in-vehicle occupant detection (VOD) systems). V2X communication enables the communication of vehicles with other entities, such as vehicles (in vehicle-to-vehicle communication, i.e., V2V), pedestrians (in vehicle-to-pedestrian communication, i.e., V2P), infrastructures (in vehicle-to-infrastructures communication, i.e., V2I), and networks (in vehicle-to-network communication, i.e., V2N) [4,5,6]. These V2X communications are based on two main wireless access technologies, i.e., the well-known and consolidated IEEE 802.11p that operates at the ITS-5.9 GHz band [7] and the Cellular-V2X (C-V2X) [8,9,10,11,12] which is a more recent technology in which V2X functionalities are widened by using 5G technology.

With the advent of these new vehicular communication technologies, people inside and nearby the connected vehicles are exposed to radiofrequency electromagnetic fields (RF-EMFs) emitted by these technologies. As evidenced in a recent survey on wireless technologies used in connected vehicles [13], research on the assessment of exposure due to V2X and automotive sensing is scarce. There exist only a few articles that addressed this topic [14,15,16]. Specifically, Tognola et al. [14] assessed the RF-EMF dose absorbed by an adult passenger inside a car equipped with V2V antennas operating at the ITS-5.9 GHz band, whereas Benini et al. [15] investigated the exposure outside the car in an adult pedestrian standing near a car equipped with the same V2V technology. In both studies [14,15], it was evidenced that the dose absorbed by the body was always below the basic restriction limits of 0.08 W/kg over the whole body, 2 W/kg in 10 g of tissues in the head and torso region, and 4 W/kg in 10 g of tissues in the limb region as recommended by the ICNIRP [17] and IEEE [18] guidelines for exposure in the 100 kHz–6 GHz range. However, to the best of the authors’ knowledge to date, nothing is known in terms of children’s exposure.

Thus, the objective of this study is to evaluate the RF-EMF exposure in children standing near a car equipped with V2V communication technology operating at the ITS-5.9 GHz band through an electromagnetic computational technique. In our previous study [15], we focused on the variability of exposure levels as a function of the distance and position of an adult pedestrian near the car. In the current study, we focused on another aspect, i.e., on the assessment of the exposure as a function of body size and age. This study builds upon the knowledge acquired in [15] for what concerns the identification of the distance and position near the car that corresponded to the worst-case exposure.

To discover whether and how the RF-EMF exposure in children varied in age and body size, four different numerical anatomical models of children of different sizes, ages, and both genders were used. Furthermore, the exposure level calculated here for the child models was then compared to that one found in similar exposure conditions in the adult model analyzed in [15].

Finally, the exposure levels obtained in children were also compared to the basic restriction limits set by the current ICNIRP [17] and IEEE [18] recommendations on EMF exposure.

## 2. Materials and Methods

### 2.1. The Exposure Scenario

**Figure 1:**
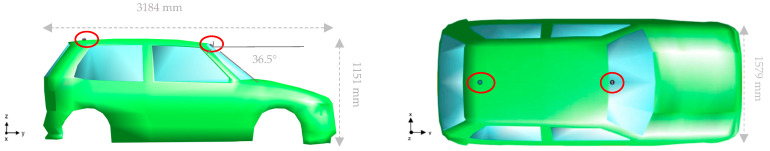
The exposure scenario from the lateral (**left panel**) and top (**right panel**) view. The red circles show the position of the V2V antennas.

Figure 1 shows the lateral view (left panel) and top view (right panel) of the exposure scenario that consisted of a 3D CAD model of a vehicle equipped with two vertical V2V antennas [15] modeled as quarter-wave monopoles [14,15,19] operating at 5.9 GHz, according to the IEEE 802.11p protocol [7]. The ground and arm of the monopoles were modeled as a Perfect Electric Conductor (PEC). Each antenna was fed with a harmonic signal at the reference input power of 1 W (i.e., 30 dBm). The antennas were mounted at two locations on the car [20,21]: one on the back roof of the car and one on the windscreen, tilted by 36.5° with respect to the horizontal plane (Figure 1). The size of the 3D CAD model of the vehicle was 1579 mm × 3814 mm × 1151 mm (Figure 1). The body of the car was modelled as PEC material, whereas the windows were modelled as glass with dielectric characteristics equal to ε_r_ = 4.82 and σ = 0.0043 S/m. The interior of the car was filled with air [22].

To investigate RF-EMF field exposure, we used the numerical anatomical models of four different children of both genders and different ages and body sizes. Table 1 lists the characteristics of the four child models. The models were taken from the Virtual population ViP2.0 (https://itis.swiss/virtual-population/virtual-population/overview/, accessed on 23 Mach 2023) of anatomical models developed from a collection of high-resolution MRI data [23]. The models resembled the anatomical characteristics of real children and included 22 different tissues with a high degree of resolution of (0.5 × 0.5 × 0.5) mm^3^ (https://itis.swiss/virtual-population/virtual-population/vip2/, accessed on 23 March 2023). To facilitate the comparison, the last row of Table 1 also shows the characteristics of the adult model ‘Ella’ investigated in [15].

**Figure 2:**
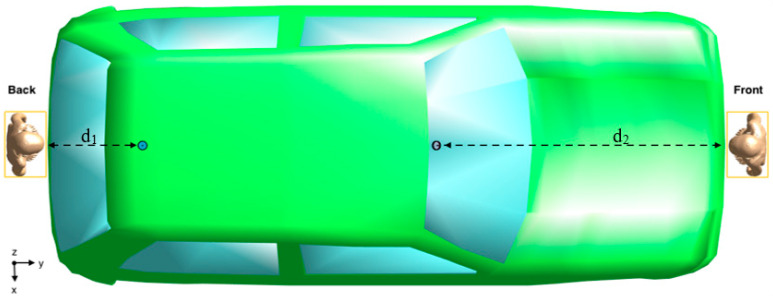
Top view of the exposure scenario with the position of one of the four child models (the ‘Thelonious’ model) at the Front and Back of the car. The yellow rectangles corresponded to the “Huygens’ Box” used to solve the electromagnetic field simulations (for details see the next section “Computational approach”). The picture also shows the distances d_1_ and d_2_ between the Huygens box and the nearest antenna (the numerical values are reported in Table 2).

**Figure 3:**
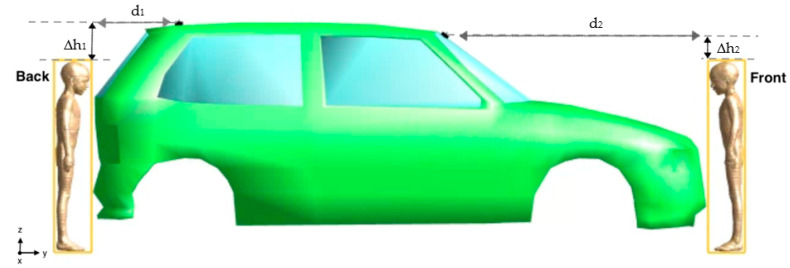
Lateral view of the exposure scenario with the position of one of the four child models (the ‘Thelonious’ model) at the Front and Back of the car. The picture shows the distances d_1_ and d_2_ (as in Figure 2) and the relative heights Δh_1_ and Δh_2_ between the topmost part of the child model and the nearest antenna (the numerical values are reported in Table 2).

Each child model was positioned once at the Front and the other time at the Back of the car. These positions were recommended by the IEEE/IEC 62704-2 [24] and supported by the finding presented in our previous study [15], where we investigated the variability of the exposure levels across different distances and positions in close proximity to the car for the ‘Ella’ model. In [15], we discovered that at the Front and Back positions near the car, the human model was exposed to the highest RF-EMF levels emitted by the two V2V antennas. Each child model was put as close as possible to the vehicle to test the worst-case exposure scenario. Figure 2 and Figure 3 show the Front and Back position near the car of one of the child models and the distances d_1_, d_2_, Δh_1_ and Δh_2_ between the model, the car, and the antennas. Table 2 reports for all the child models the numerical values of the relative heights Δh_1_ and Δh_2_ between the topmost part of each model and the nearest antenna and the horizontal distances d_1_ and d_2_ between each human model and the nearest antenna. As for Δh_1_ and Δh_2_, a positive sign indicates that the top of the child model is below the antenna height. It is important to note that the numerical values of Δh_1_, Δh_2_, d_1_, and d_2_ are not the same for all the child models because they depend on the different body sizes and heights of the models. For comparison, in the last row of Table 2 are also reported the relative heights and the distances from the antenna of the adult model Ella investigated in [15].

A total of eight different exposure simulations were done, corresponding to four child models evaluated at two positions near the car.

**Table 2:** The relative heights between the topmost part of each human model and the antenna at the back (Δh_1_) and front (Δh_2_) of the car and the distances d_1_ and d_2_ from the nearest antenna. The values for Ella were taken from [15].

Human Model	Δh_1_ (mm)	Δh_2_ (mm)	d_1_ (mm)	d_2_ (mm)
Roberta	+319	+244	539	1623.2
Thelonious	+241.7	+166.6	547	1624.6
Eartha	+46.7	−28.4	540	1624
Dizzy	+12.7	−62.4	540	1624
Ella	−212.3	−287.4	536	1608

### 2.2. Computational Approach

Electromagnetic field simulations were performed to calculate the EMF generated by the antennas in the exposure scenario. The simulations were done by using the Finite-Difference Time-Domain (FDTD) solver of Maxwell’s equations as implemented in the software simulator Sim4Life [25]. The FDTD method is one of the most popular, well-validated and consolidated numerical approaches used to solve computational electromagnetic problems. Briefly, the FDTD method involves both a spatial and temporal discretization of the electric and magnetic fields within the spatial computational domain under test. The spatial computational domain has to be limited by proper boundary conditions to avoid possible EMFs reflection back into the domain. In our simulations, a perfectly matched layer (PML) absorbing condition was assumed at the boundaries of the computational domain.

The computational domain in our simulations comprised the CAD model of the vehicle, the two V2V antennas, and one child model positioned in one of the two positions at a time. We discretized the computational domain with a non-uniform grid of 1 mm for the back antenna and with a finer grid of 0.2 mm for the front antenna to reproduce its tilted shape as accurately as possible. The biological tissues of the child models were discretized with a maximum step of 0.86 mm for the skin and 0.632 mm for the eye tissues (cornea, vitreous humor, sclera, and lens). The dielectric properties of the tissues of the children’s models were set according to the literature data [26,27,28].

The number of cells resulting from the discretization of the computational domain ranged from 3.3 Gcells for the simulation with the shortest model (Roberta) to 4 Gcells with the tallest one (Dizzy). Such a high number of cells implies a great effort in terms of computational time and memory consumption. Thus, we applied the so-called “Huygens’ Box” approach [29,30], which is a two-step electromagnetic computational approach to make the simulations more computationally affordable [15,31]. In the first step of the Huygens’ Box approach, the EMF calculation is done considering the exposure scenario described above but without the human model. In this step, the procedure calculated the electric and magnetic surface currents on the Huygens’ Box, which is a 3D rectangular-shaped area around the child model. In the second step, the calculation domain includes only the human model, which is put inside the Huygens’ Box. The surface currents calculated in the first step were used in the second step as excitation sources to generate the same fields as the ones originally generated by the two V2V antennas. The Huygens’ Box, which corresponded to the yellow rectangular areas in Figure 2, had different dimensions that depended on the different sizes of the human models. Table 3 reports the geometrical dimension of the Huygens’ Box for each children’s model.

### 2.3. Exposure Assessment

The assessment of the dose of EMF absorbed by the child models due to the two V2V antennas was performed by investigating the Specific Absorption Rate (SAR) over the whole body (wbSAR [W/kg]), which is the ratio between the EMF power absorbed by the whole body over the total mass of the body and the tissue-specific SAR averaged over 1 g (SAR_1g_ [W/kg]) and 10 g (SAR_10g_ [W/kg]) of body tissue. We decided to calculate the SAR only for the most superficial tissues and organs, i.e., the skin and the eyes, because, at the frequency of 5.9 GHz used in vehicular communications, the radiation does not penetrate into more profound subcutaneous tissues than the skin and eyes [32,33]. As recommended by the ICNIRP, for the skin, we averaged the SAR over 10 g of tissue. Specifically, we calculated the SAR_10g_ over the skin of the whole body and over the skin of the head and genital areas, which are the body’s most sensitive areas to RF-EMF exposure [17]. Instead, for the eyes, because of their small mass, we averaged the SAR over 1 g of tissue. Specifically, we calculated the SAR_1g_ in each of the different eye tissues included in the models, i.e., the cornea, sclera, lens, and vitreous humor.

As for the eyes, all models apart from the Dizzy model included four eye tissues (cornea, sclera, lens, and vitreous humor); the Dizzy model included only two tissues, i.e., the vitreous humor and lens. Therefore, in the interpretation of the results, this difference will be taken into account. A descriptive statistic showing the maximum, the median, and the skewness of the SAR_1g_ and SAR_10g_ distributions in the different tissues and body regions were computed.

Finally, the children’s SAR values were compared to the ones of the adult model ‘Ella’ previously investigated in [15] and to the exposure limits recommended in the ICNIRP [17] and IEEE guidelines [18].

## 3. Results

We remind the reader that all the SAR values reported in this section were calculated by feeding both antennas simultaneously with 1 W (i.e., 30 dBm). This is the worst-case exposure condition because, in real scenarios, the V2V antenna system typically employs adaptive power control, so the typical real forward power might be less than 1 W.

Table 4 shows the wbSAR values across the children’s models and the two positions near the car. The child models in Table 4 are listed from the shortest (Roberta) to the tallest one (Dizzy). To facilitate the comparison, the bottom row of Table 4 also displays the wbSAR of the adult model Ella investigated in [15]. In all the child models, the wbSAR at the Back position was always lower than that at the Front position. The wbSAR at the Front position was almost the same across the children. At the Front position, the children and the adult showed almost similar values of wbSAR, ranging from 0.15 to 0.18 mW/kg. Vice versa, at the Back position, the difference in the wbSAR values across the human models was very pronounced and ranged from 0.02 mW/kg in the shortest child model (Roberta) up to 0.19 mW/kg in the tallest model (Ella). Indeed, when the human model was near the back antenna, the increase in the model height resulted in a decrease of the relative height Δh_1_ from the back antenna, going from +319 mm in Roberta (the shortest child) to −212.3 mm in the adult Ella (Table 2). The decrease of Δh_1_ resulted in an increase in the models’ upper body area exposed to the radiation of the back antenna, reaching the maximum wbSAR in the tallest model. On the contrary, at the Front position, thanks to the tilted configuration of the frontal antenna, the children and adult models were always entirely exposed to the same degree of radiation, regardless of their height. Differently from the children, the wbSAR of the adult model (Ella) did not significantly change between the two positions near the car because the model is tall enough to be radiated also when it is at the back of the car.

**Figure 4:**
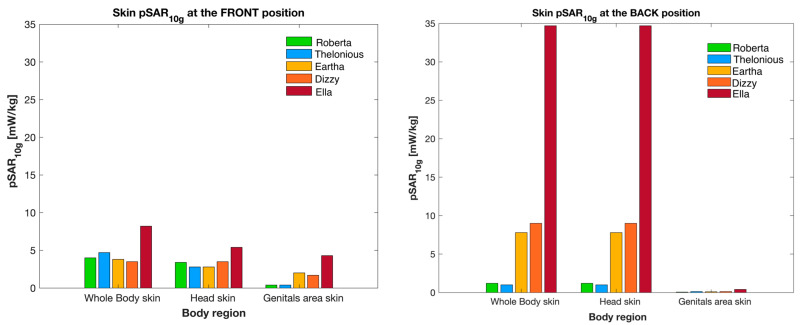
pSAR_10g_ in the different body regions of the skin for the children and adult models at the Front (**left panel**) and Back (**right panel**) positions near the car.

**Figure 5:**
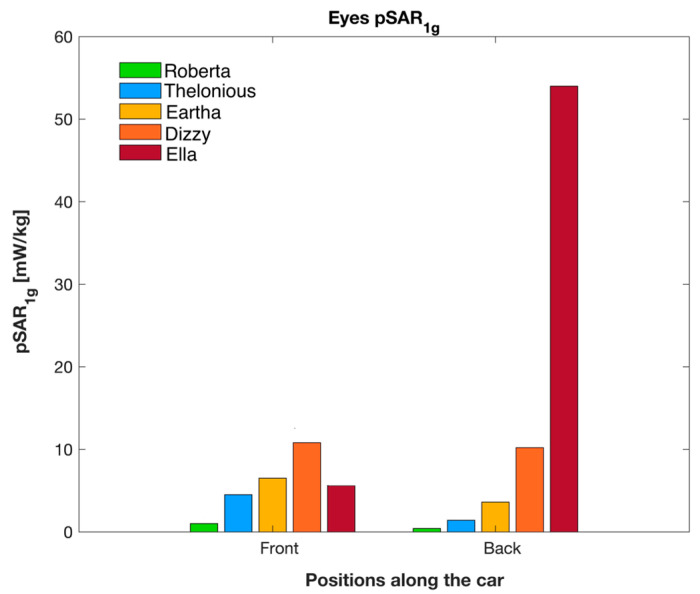
pSAR_1g_ of the eyes in the Front and Back positions near the car for the children and adult models.

Figure 4 shows the maximum peak SAR values averaged over 10 g (pSAR_10g_) of the skin of the whole body, the head, and the genitals area, while Figure 5 displays the maximum peak SAR values averaged over 1 g (pSAR_1g_) of the eyes. As in Table 4, to facilitate the comparison, Figure 4 and Figure 5 also show the pSAR values obtained with the adult model [15].

Consistently with the wbSAR values of Table 4, at the Front position, the pSAR_10g_ values in the skin (Figure 4) did not significantly change across the children; also, in this position, the children and the adult had almost similar pSAR_10g_ values. Vice versa, at the Back position, the pSAR_10g_ values of the skin of the children were significantly lower than in the adult, especially for the shortest children, i.e., Roberta and Thelonious, for which pSAR_10g_ was nearly negligible. At the Back position, the exposure of the whole body and the head region of children increased with the child’s height because the relative height Δh_1_ between the back antenna and the head region decreased with the increase of the model height (Table 2). Considering both the Front and Back positions, the pSAR_10g_ value in the skin of the whole body and in the head region ranged from 1.04 mW/kg to 9 mW/kg of the children, while in the adult, the pSAR_10g_ value obtained in the whole body and head region reached the maximum of 34.70 mW/kg (Figure 4). Instead, the pSAR_10g_ value in the skin of the genitals area was very small for both the children and the adults, being almost negligible at the Back position where the lower body region is scarcely exposed to the radiation of the back antenna.

As for the eyes (Figure 5), it is noteworthy to observe that the pSAR_1g_ values in the children were generally higher at the Front position than at the Back position, ranging from 0.40 mW/kg in the shortest child (Roberta) to 10.80 mW/kg in the tallest one (Dizzy). This was because, at the Front position, the eyes of the children were always well exposed to the radiation of the frontal antenna; in this position, the higher the child, the higher the exposure because the antenna-to-eyes distance decreased with the child’s height. In contrast, the eyes of the adult had a lower exposure at the Front (5.61 mW/kg) than at the Back position (54 mW/kg) because the distance Δh_1_ between the eyes and the back antenna is smaller than the distance Δh_2_ between the eyes and the front antenna (|Δh_1_| = 212.3 mm and |Δh_2_| = 287.4 mm (Table 2)).

**Figure 6:**
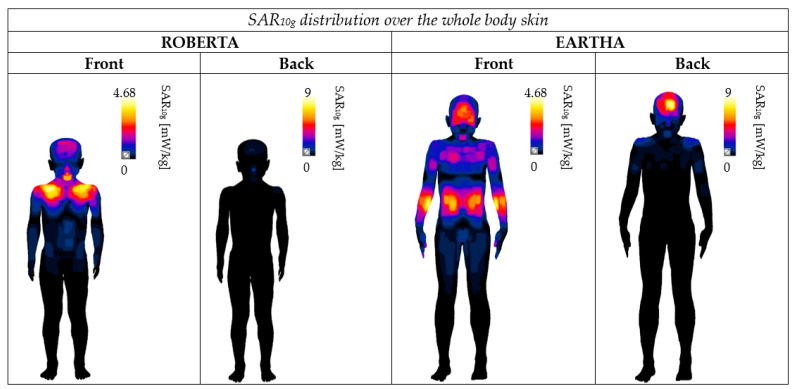
SAR_10g_ distribution on the skin of the whole body in Roberta (the shortest child) and Eartha (one of the tallest children) at the Front and Back positions. For both models and positions, the SAR_10g_ distribution was normalized to the maximum SAR found among the children, i.e., 4.68 mW/kg at the Front position and 9 mW/kg at the Back position.

Table 5 reports for each human model the sites on the body skin characterized by the maximum exposure (i.e., characterized by the pSAR_10g_ value shown in Figure 4). When the models were in the Back position, the maximum exposure in the whole body was always located at the head, namely at the nose and forehead. Vice versa, at the Front position, the maximum exposure in the whole body was not always at the head level but, except for Dizzy, tended to move from the top (the shoulders) to the bottom (the hands) as the model height increased. Specifically, for the head, the sites of the maximum exposure across the Front and Back positions were the chin, the forehead, and the nose. As an example, Figure 6 shows the SAR_10g_ distribution on the skin of the whole body in the shortest (Roberta) and the tallest (Eartha) child models. From Figure 6, it is possible to observe how the location of the peak SAR value changes as the height of the model increases. Finally, for the genital area (Table 5), only for two models (Thelonious and Dizzy) in the Back position, the maximum exposure was located right at the genital organ (i.e., in the penis); in all the other models, the maximum was located elsewhere, such as at the upper thighs or the waist.

**Table 5:** Location of the maximum exposure in the skin of the whole body and at the head and the genital area for the Front and Back positions of the models near the car.

Human Model	Body Region	pSAR_10g_ Site	
		Model Position: Front	Model Position: Back
Roberta	Whole-body	shoulder	nose
	Head	chin	nose
	Genitals area	upper thigh	upper thigh
Thelonious	Whole-body	arm	forehead
	Head	forehead	forehead
	Genitals area	upper thigh	penis
Eartha	Whole-body	forearm	forehead
	Head	forehead	forehead
	Genitals area	waist	waist
Dizzy	Whole-body	nose	forehead
	Head	nose	forehead
	Genitals area	waist	penis
Ella	Whole-body	hand	nose
	Head	nose	nose
	Genitals area	waist	waist

**Figure 7:**
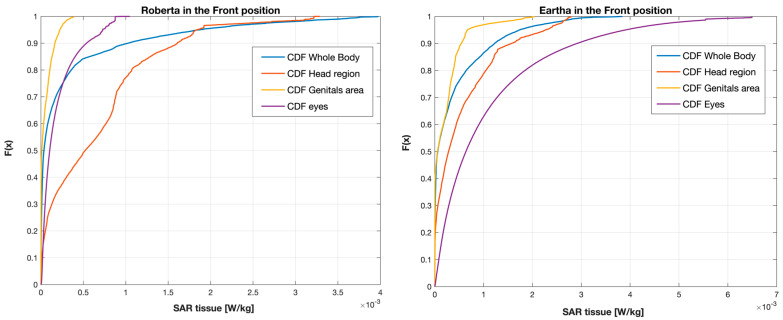
CDF of each tissue region investigated in Roberta (**left**) and Eartha (**right**) at the Front position. The *x*−axis corresponds to the SAR values averaged over 10 g (SAR_10g_) for the skin of the whole body, head region and genital area, and 1 g (SAR_1g_) for the eyes.

To further delve into the study of the exposure, Figure 7 reports some examples of SAR distributions in terms of Cumulative Distribution Function (CDF) for each tissue region investigated at the Front position in Roberta and Eartha (the same children illustrated in Figure 6). In order to conduct a more comprehensive and quantitative analysis of the exposure, Table 6 reports the maximum (also illustrated in Figure 4 and Figure 5 as pSAR_10g_ for the skin and pSAR_1g_ for the eyes, respectively), the media, and the skewness of the distribution of the SAR_10g_ and SAR_1g_ in the skin and eyes of the children and the adult model Ella. Generally, it can be noticed that the median values were two orders of magnitude lower than the maximum values for all the human models and both positions near the car. Also, for both the children and the adult, the SAR_10g_ and SAR_1g_ distributions had a positive skewness, meaning that there was a greater density of SAR values towards the lower range of exposure levels.

The median value of SAR_10g_ of the skin of the whole body ranged from 0.01 to 0.06 mW/kg across the two positions and all models. As already observed for the maximum exposure (see Figure 4), the head was the body region with the greatest exposure, with a median value ranging from 0.05 mW/kg in the adult in the Front position to 0.60 mW/kg in the tallest child (Dizzy) in the Back position; the dose absorbed at the genital region was always negligible with very low median values near to zero. As already observed with the maximum, the dose absorbed by the smallest children (i.e., Roberta and Thelonious) at the whole body was higher at the Front position than at the Back, while for the tallest child and the adult, the dose absorbed was higher at the Back position. This was because in the Back position, the upper body regions were more exposed to the radiation in the tallest child and adult (because of their lowest Δh_1_) rather than in the shortest child, which instead had a greater exposure at the Front.

The dose absorbed by the eyes was generally higher than that of the whole body for both the children and the adult, with a median value ranging from 0.03 mW/kg to 3 mW/kg across the two positions and all models. This is because the eyes were generally entirely well exposed to the radiation, especially in the Front position. For the children, the median value of the dose of exposure at the eyes was higher when the model was at the Front position of the car. Vice versa, in the adult, the median value of the exposure dose in the eyes was higher at the Back position.

It can also be observed from Table 6 that the maximum and the median value of the exposure in the eyes progressively increased with the child’s height because the taller the child, the closer the antenna was to the eyes. The greatest exposure in the eyes was observed in Dizzy, which was the tallest among the child models. Although the Dizzy model includes only two (the vitreous humor and lens) of the four eye tissues of the other models, its SAR values in the eyes were in line and even greater than those calculated in the other children’s models. It is noted that the Dizzy model includes the vitreous humor, which is the eye tissue with the greatest weight and the highest dielectric properties due to its 99.7% content in water [34]. These characteristics make the vitreous humor the most relevant tissue for SAR characterization in the eyes. As such, we can safely conclude that the SAR values we obtained in the eyes of Dizzy were not underestimated.

## 4. Discussion

This study investigated RF-EMF exposure in children in the ITS-5.9 GHz vehicular connectivity exposure scenario. This study calculated and compared the exposure levels in anatomical models of children with the ones of an adult female model previously investigated in [15] in the same exposure setup. Exposure was evaluated through numerical dosimetry in four anatomical models of children of different body sizes, ages, and both genders, namely in the Roberta (female, 5 years old), Thelonious (male, 6 years old), Eartha (female, 8 years old), and Dizzy models (male, 8 years old). The exposure of each child model was simulated and evaluated at two different positions—Front and Back—near a car equipped with two V2V antennas.

In all the models and both positions, it was found that the RF-EMF dose was absorbed mainly in the most superficial tissues and organs, i.e., in the skin and eyes. The body region with the highest RF-EMF absorption was found to be in the upper body, specifically, in the shoulder and arm for the shortest children (i.e., Roberta and Thelonious) at the Front position of the car and in the head region, i.e., the nose and forehead, for the tallest children (i.e., Eartha, Dizzy) and the adult model when they were at the Back of the car.

The dose absorbed by the children was lower than that of the adult. The difference between the children and the adult was more pronounced for the exposures at the Back of the car. Overall, considering the exposures across the Front and Back positions together, the dose absorbed by the whole body (wbSAR) ranged from 0.17 to 0.19 mW/kg for the adult and from 0.02 to 0.18 mW/kg for the children. For the tissue-specific SAR for both the children and the adult, the greatest exposure was observed in the skin. Among the children, the maximum absorption of 9 mW/kg was found at the skin of the head region of the tallest child (i.e., Dizzy). This latter value was much lower than the maximum exposure of 34.70 mW/kg found in the adult (Ella). Similarly, also for the eyes, the children exhibited lower exposure levels than the adult. The highest value of 10.80 mW/kg was found again in the tallest child, whereas the maximum exposure in the adult was 54 mW/kg. The exposure of the lower body region, i.e., at the genital area, was negligible for both the children and the adults, especially at the Back position.

For all the children and positions near the car, the dose absorbed by the whole body and by the different body regions was well below the basic restriction limits recommended by the ICNIRP and IEEE guidelines for the exposure of the general public in the 100 kHz–6 GHz range, which are 0.08 W/kg for the whole body, 2 W/kg in 10 g of tissue of the head and torso regions, and 4 W/kg in 10 g of tissues of the limb region [17,18]. As described above, the highest dose of exposure in the children was in the eyes (equal to 10.80 mW/kg). It is worthwhile to remind that we calculated the dose in the eyes over a 1 g mass of tissues instead of using a 10 g mass as we did for the skin. For the eyes, we preferred to use the 1 g mass instead of the 10 g mass as indicated in the current ICNIRP and IEEE guidelines because of the small mass of the eyes. As such, the compliance of the dose calculated by us in the eyes of the ICNIRP and IEEE basic restriction limits can be done only at a qualitative level. Indeed, since the calculation of the dose absorbed by a 1 g tissue usually is greater than the value that would be obtained with a 10 g mass, we can conclude that the dose of 10.80 mW/kg estimated in our study on a 1 g mass would be in any case well below the ICNIRP and IEEE safety limit of exposure of 2 W/kg in a 10 g mass.

Our SAR levels were obtained by driving both antennas with 1 W of transmit power. If we scale our SAR values to the maximum transmit power of 33 dBm (i.e., 1.99 W) for each of the two V2X communication antennas, as allowed in the EU and the US for non-government services [7], the SAR levels would be almost doubled. Nevertheless, also in this latter circumstance, the dose absorbed would remain below the limits recommended by ICNIRP and IEEE [17,18].

Considering that the dielectric properties of the children and adult tissues are the same, the differences in the exposure levels within the children and between the children and the adult models were most probably due to a combination of two factors, namely the different anatomical characteristics of the models (such as the height) and to the position of the models near the car. Both factors have an impact on the degree to which each model was radiated by the nearest antenna. For the anatomical characteristics, height seems to play an important role, mainly for the exposure at the Back of the car. In this latter position, the field emitted by the rear antenna propagated mostly in a horizontal plane parallel to the ground. As such, this field could radiate only those models (the tallest ones) that were at the same or at a higher level than the antenna. At the Back position, the increase of the model height resulted in an increase in the upper body regions that could be directly exposed to the radiation. As a result, the whole body and the tissue-specific SAR at the Back position gradually increased as the model’s height increased. Vice versa, at the Front position, where the field of the antenna propagates more towards the ground (because the antenna is tilted), all models are well exposed, and the effect of the model height was less relevant.

As described in the Results, at the Back position, there was a relevant difference in the SAR of the smallest child and the adult. In particular, the whole-body SAR was 0.02 mW/kg in the smallest child (Roberta) and 0.19 mW/kg in the adult; the maximum of the SAR of the adult was almost four times higher than that of the children for the skin and 5.3 times higher for the eyes. On the contrary, at the Front position, the SAR values of the whole body and the skin of the children and the adult were more similar. For the eyes, the local SAR increased with the child’s height, reaching the maximum value of 10.80 mW/kg for the tallest child at the Front position. For the adult, the maximum SAR of the eyes (54 mW/kg) was observed at the Back position.

The statistical analysis revealed that the distributions of the SAR values in the skin and the eyes were positively skewed, meaning that most of the values were distributed in the lower range of exposure levels. The median value of the SAR in the skin of the whole body ranged from 0.01 mW/kg to 0.06 mW/kg in the children and from 0.03 mW/kg to 0.06 mW/kg for the adult.

To provide a more comprehensive picture of the RF-EMF exposure in the vehicular connectivity scenario, it is worthwhile to compare the SAR values obtained here for the child models with the ones obtained in [14] for an adult model placed inside a vehicle equipped with the same V2V antennas used in the current study. The authors in [14] mounted four antennas symmetrically at the front/rear roof and left/right mirrors of the car. Each antenna was fed with the maximum transmitted power allowable in the US for government services of 44.8 dBm (i.e., 30.2 W) [7]. If we scale our SAR levels by feeding each of the two V2V antennas with the maximum input power of 30.2 W, the highest wbSAR value obtained in the tallest child at the Front position, i.e., 0.18 mW/kg, would become 5.43 mW/kg. This last value is lower than the wbSAR values obtained in [14] of 8.33 mW/kg, most likely because of the higher number of antennas used in [14] (four antennas) compared to our case (two antennas).

Furthermore, it is expected that the exposure scenario investigated in [14] would induce higher SAR values than in our scenario also because of the shorter distance between the human model and the nearest antennas, namely 0.1–0.5 m in [14] and 0.5–1.6 m in our scenario. Similarly to our study, the highest local SAR value observed in [14] was located in the skin. However, while in [14] the maximum peak was located in the skin of the head region, in our case, this holds true only for the tallest children (i.e., Eartha and Dizzy) because for the smallest ones (i.e., Roberta and Thelonious) the maximum SAR was located in the shoulder and arm. In particular, comparing the local SAR values, the pSAR_10g_ found in [14] was 1581 mW/kg, which is much higher than the maximum pSAR_10g_ value found by us among the children, i.e., 271.80 mW/kg in the tallest child at the Back of the car when the antennas were fed with 30.2 W. This relevant difference is most likely due to the shorter distance between the human model and the nearest antennas [14].

## 5. Conclusions

This is the first study that investigated the dose of RF-EMF absorbed in V2V communication scenarios by children pedestrians. This is an important topic as children are likely to face a longer period of RF-EMF exposure compared to present-day adults. The current study applied a computational method, i.e., the FDTD, to calculate the dose absorbed in anatomical models of children of different body sizes and compared the values thus obtained with those calculated by a previous study [15] in an adult model. For both the children and the adult models, the highest exposure levels were found in the upper body region of the skin (head, shoulders, arms) and eyes. The adult had higher SAR values compared to those of the children. The exposure in children was influenced by their height and relative position from the car because, for certain positions near the car (the Back position in our scenario), the smallest children might be scarcely radiated by the nearest antenna. All the SAR levels are well below the limits recommended by the ICNIRP and IEEE guidelines [17,18].

To fully assess the exposure levels generated in children by the ITSs technologies in the connected vehicle, also the technologies for automotive sensing have to be considered in the forthcoming studies, as well as the other V2X communication technologies, i.e., V2I, V2P, and V2N. Furthermore, as the next step, it would be interesting to investigate the time dependence of the exposure levels to provide a more complete investigation.

## Figures and Tables

**Table 1:** The characteristics of the child and adult models (https://itis.swiss/virtual-population/virtual-population/overview/, accessed on 23 March 2023). BMI—Body Mass Index (kg/m^2^).

Name	Sex	Age (Year)	Height (m)	Weight (kg)	BMI (kg/m^2^)
Roberta	female	5	1.1	17.6	14.8
Thelonious	male	6	1.16	18.6	13.8
Eartha	female	8	1.36	29.9	16.2
Dizzy	male	8	1.37	25.3	13.5
Ella	female	26	1.63	57.3	21.6

**Table 3:** Dimension of the Huygens’ Box along the three axes *x*, *y*, *z*, for each child model.

Human Model	Huygens Box (mm)
Roberta	366 × 241 × 1136
Thelonious	396 × 246 × 1211
Eartha	481 × 273 × 1419
Dizzy	439 × 266 × 1461

**Table 4:** The wbSAR across the different human models at the Front and Back positions near the car. The values for Ella were taken from [15].

Human Model	wbSAR (mW/kg)	
	Front	Back
Roberta	0.15	0.02
Thelonious	0.15	0.03
Eartha	0.17	0.10
Dizzy	0.18	0.14
Ella	0.17	0.19

**Table 6:** Descriptive statistics of the SAR_10g_ and SAR_1g_ distribution of the skin and the eyes for the different human models at the two positions near the car. The maximum values were the same as displayed previously in Figure 4 and Figure 5 as pSAR_10g_ and pSAR_1g_, respectively_._ The values of Ella were taken from [15].

Body Region	Parameters	Roberta		Thelonious		Eartha		Dizzy		Ella	
		Front	Back	Front	Back	Front	Back	Front	Back	Front	Back
Skin of the Whole Body	- SAR_10g_ maximum (mW/kg)	4.00	1.18	4.68	1.04	3.83	7.80	3.54	9.00	8.20	34.70
	- SAR_10g_ Median (mW/kg)	0.04	0.01	0.04	0.01	0.06	0.01	0.05	0.01	0.06	0.03
	- Skewness	3.00	4.40	3.57	3.92	2.32	5.70	2.38	5.54	2.50	5.27
Skin of the head region	- SAR_10g_ maximum (mW/kg)	3.37	1.18	2.84	1.04	2.80	7.80	3.54	9.00	5.40	34.70
	- SAR_10g_ Median (mW/kg)	0.52	0.09	0.20	0.10	0.30	0.30	0.40	0.60	0.05	0.30
	-Skewness	1.37	1.64	1.95	1.00	1.54	1.78	1.31	1.4	2.14	2.19
Skin of the genital region	- SAR_10g_ maximum (mW/kg)	0.38	0.05	0.39	0.12	2.04	0.10	1.71	0.12	4.30	0.40
	- SAR_10g_ Median (mW/kg)	0.02	0	0.02	0	0.06	0	0.08	0	0.02	0
	-Skewness	1.77	1.43	1.16	1.00	2.77	1.45	2.80	1.38	1.88	2.10
Eyes	- SAR_1g_ maximum (mW/kg)	1.05	0.40	4.47	1.37	6.50	3.60	10.80	10.20	5.61	54.00
	- SAR_1g_ Median (mW/kg)	0.10	0.03	0.50	0.10	0.60	0.30	1.30	1.30	0.30	3.00
	- Skewness	1.63	1.73	1.67	1.72	1.82	2.14	1.56	1.61	2.23	2.20

## Data Availability

Not applicable.
